# There Is Always Another Way! Cytomegalovirus’ Multifaceted Dissemination Schemes

**DOI:** 10.3390/v10070383

**Published:** 2018-07-20

**Authors:** Joseph W. Jackson, Tim Sparer

**Affiliations:** Department of Microbiology, University of Tennessee Knoxville, Knoxville, TN 37996, USA; jjacks99@vols.utk.edu

**Keywords:** viral dissemination, innate immune cells, cytomegalovirus, pathogenesis, chemokines, Fenner hypothesis, neutrophils, monocytes

## Abstract

Human cytomegalovirus (HCMV) is a β-herpes virus that is a significant pathogen within immune compromised populations. HCMV morbidity is induced through viral dissemination and inflammation. Typically, viral dissemination is thought to follow Fenner’s hypothesis where virus replicates at the site of infection, followed by replication in the draining lymph nodes, and eventually replicating within blood filtering organs. Although CMVs somewhat follow Fenner’s hypothesis, they deviate from it by spreading primarily through innate immune cells as opposed to cell-free virus. Also, in vivo CMVs infect new cells via cell-to-cell spread and disseminate directly to secondary organs through novel mechanisms. We review the historic and recent literature pointing to CMV’s direct dissemination to secondary organs and the genes that it has evolved for increasing its ability to disseminate. We also highlight aspects of CMV infection for studying viral dissemination when using in vivo animal models.

## 1. Introduction

Human cytomegalovirus (HCMV) is a ubiquitous beta-herpesvirus with a 50–90% seroprevalence rate in the adult human population [1]. While HCMV infection is usually asymptomatic, severe disease can result from primary infection or viral reactivation from latency in immune compromised hosts [2,3]. HCMV infection in immunocompromised persons can result in interstitial pneumonia, gastroenteritis, retinitis, organ transplant rejection, or death [4,5]. HCMV is also a leading cause of congenital disease [6,7]. Upon in utero infection, the child can exhibit microcephaly or severe sequelae, or both, including hearing loss, mental retardation, and learning disabilities [8,9,10]. Whether infection is due to primary infection or reactivation, HCMV disease is caused through viral dissemination and inflammation. Therefore, understanding the basics of CMV’s lifecycle will provide new avenues for interventions, which could limit HCMV diseases.

**Figure 1:**
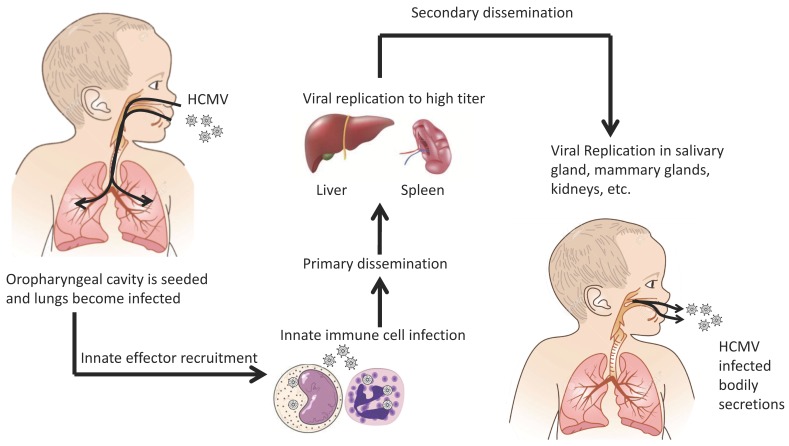
Overview of human cytomegalovirus (HCMV) dissemination.

HCMV uses several routes to spread within the populous. Vertical transmission occurs through transplacental and intrapartum transmission [10,11,12] through breast feeding from an infected mother to the child [13,14,15,16]. Horizontal transmission occurs through organ transplantation of an infected organ or contact with infected bodily secretions (i.e., saliva, breast milk, urine, etc.) [17,18,19,20]. Following initial exposure, HCMV is thought to undergo a brief leukocyte-associated viremia during which organs such as the lung, spleen, and liver become seeded and productively infected [2,3]. This sequence of events has historically been termed primary viral dissemination. Following primary dissemination, the virus undergoes a sequential dissemination (i.e., secondary dissemination) in which HCMV infects tissues such as the salivary glands, breasts, and kidneys [3]. Because the virus is spread via bodily fluids, it is presumed that HCMV targets these organs in order to infect new hosts. Figure 1 is the historical overview of HCMV dissemination. In an immunocompetent person the infection is contained, but like all herpes viruses, the virus remains latent for the host’s lifetime. Because a substantial portion of the human population is infected with HCMV, this leaves a large pool of people with latent infections. These individuals are potentially susceptible to viral morbidity if immunocompromised and HCMV reactivates. How, when, and why HCMV reactivates is unknown but for a complete review on HCMV latency see Sinclair and Poole [21].

Dr. Frank Fenner, studying mouse pox, postulated that during a viral infection the virus would replicate at the infection site, then disseminate to the regional lymph nodes, followed by replication in blood filtering organs. These organs would then produce large quantities of virus that results in disease [22,23]. This longstanding hypothesis assumes that virus undergoes a gradual increase in viral burden throughout the host, which eventually leads to disease. Data from mouse CMV (MCMV) models point to a different in vivo scenario. During the course of an MCMV infection, the virus undergoes a biphasic viremia in which the virus briefly appears in circulation, disappears, and eventually reappears in the blood [24]. Another CMV characteristic which contradicts Fenner’s model, is that CMV infection is cell mediated and cell-free virus has little to no effect on the course of infection [25,26,27,28]. However, the exact mechanism by which HCMV travels through the body is unknown. This is complicated by HCMV’s multifaceted dissemination and the limitation of studying HCMV in vivo. Due to the latter, we will rely on discoveries in animal models to uncover mechanisms for CMV dissemination. HCMV disease is linked to dissemination and inflammation; therefore, if we understand viral dissemination it could help to understand HCMV pathogenesis. In this review, we will focus on key events and influential cell types in cytomegalovirus dissemination from both HCMV and small animal models.

## 2. Transmission and Initial Infection

HCMV infection is dependent on direct contact with infected bodily secretions. HCMV is shed in urine, breastmilk, and genital secretions in order to transmit to a new host [1]. It is presumed that most individuals acquire HCMV orally [29,30]. A rhesus model of CMV demonstrated that rhesus CMV oral infection leads to infection and subsequent transmission to new hosts [31]. This begs the questions: What is the primary infection site? Is it the oral cavity, the lungs, or the gut? Farrell et al. compared intranasal and oral infection models using MCMV [32]. They demonstrated that MCMV infection in either case resulted in an upper respiratory tract infection and not infection of the mouse gut. This points to intranasal infection as a more natural route for CMV infections.

After the virus breaches the host’s external barriers, it enters a host cell and begins replication. This is the initial step in viral dissemination; MCMV directly infects alveolar macrophages and type 2 alveolar epithelial cells after intranasal inoculation [33]. By extrapolation, these cell types are potentially the initial cells infected following HCMV transmission. HCMV entry into epithelial cells and macrophages is mediated by endocytosis and the subsequent pH-dependent fusion with the endosomal membrane. This is facilitated by the viral envelope glycoproteins gB, gH/gL/gO, and the pentameric complex gH/gL/UL128, UL130, UL131A [34,35,36,37,38]. Upon entry into the epithelial cell, the virus undergoes its lytic cycle, generating infectious viral progeny that infect other susceptible cell types such as fibroblasts, endothelial cells, dendritic cells, and other innate immune cells including alveolar macrophages [39,40]. During this initial stage, the virus is spread locally either by cell-free virus or via cell-to-cell spread [41]. Cell-to-cell spread is mediated in part by the HCMV gene *US28* [42]. This mechanism of spread requires direct contact between an infected cell and an uninfected cell. Interestingly, human clinical isolates do not release cell-free infectious progeny, but are still capable of efficient spread throughout a monolayer [43]. This hints that in vivo, HCMV prefers cell-to-cell spread [43,44]. As we will discuss, many of the innate immune cells which aid in CMV dissemination are efficiently infected via cell-to-cell infection but not with cell-free virus [45,46].

## 3. Cell-Mediated Dissemination

When HCMV spreads to innate immune cells, the second stage of HCMV viral dissemination commences (i.e., systemic spread). Endothelial cells (ECs) influence cell-to-cell spread [45,46]. Naïve innate immune cells (i.e., monocytes or neutrophils) are readily infected when migrated across an infected EC layer [47]. Additionally, infected ECs encourage the adherence of innate immune cells to the endothelium by increasing expression of adhesion molecules such as ICAM-1, vCAM-1, and others [48,49]. The increase in adhesion molecules increases the interactions between naïve monocytes or neutrophils, or both, and infected ECs, thereby increasing the likelihood of cell-mediated infection [48]. HCMV infection of ECs also increases vascular permeability of the endothelium, which in turn increases contact between innate immune cells and ECs [48]. This leads to increased HCMV infection of these cells. Cell-mediated infection of monocytes and neutrophils is dependent on the presence of a functional pentameric complex (i.e., gH/gL/UL128, UL130, UL131A). HCMVs deleting these genes are unable to enter ECs and by extension unable to be transferred to monocytes or neutrophils [47]. This reiterates that without viral entry, the dissemination process is crippled.

For infection of neutrophils, Gerna et al. proposed a membrane fusion between the neutrophil and the infected ECs [50]. The membranes fuse generating micro pores between the two cells. These pores will allow the virus to be shuttled from the infected endothelial cell into the neutrophil. This mechanism of viral acquisition is similar to trogocytosis, a process through which intracellular bacteria spread from cell to cell [51]. This is one of the roles that ECs play in dissemination [52,53,54]. Infected ECs may also play a more direct role in dissemination. Infected ECs can detach from the vasculature and enter the blood stream. These detached ECs are referred to as giant endothelial cells, which are capable of productive viral replication [53,54]. They can potentially transfer virus to uninfected organs within the narrow venules where cell-to-cell contact is more prevalent. However, the MCMV mouse infection model did not show direct EC involvement during dissemination [25]. In the second stage of HCMV viral dissemination, innate immune cells could have a role, but in what capacity these cells contribute to hematogenous spread of HCMV is still controversial.

HCMV viremia is mostly cell associated [3]. HCMV DNA has been found in serum and plasma of infected transplant recipients, but these are highly fragmented genomes implying that they are not infectious virions [55,56]. In support of cell-associated viremia, depletion of leukocytes from blood products derived from seropositive donors prior to blood transfusion prevents HCMV transfer [57,58]. In addition, there are numerous reports indicating that peripheral blood leukocytes harbor infectious HCMV [59,60,61,62,63]. These studies demonstrated that infectious virus was able to be isolated from both the mononuclear and polymorphonuclear (PMN) factions. This led to the hypothesis that CMVs use both monocytic cells as well as PMNs to disseminate throughout the body. In order for these cells to play any part in dissemination the innate immune cells must travel to the primary infection site, become infected, and then leave to infect other tissues.

In general, viral infection leads to an influx of cells such as monocytes and PMNs at the infection site, which could provide additional targets to aid viral dissemination. However, this response is amplified during HCMV infection. CMVs have evolved to manipulate the immune system for its benefit. The plethora of immunomodulatory proteins that HCMV encodes is outside of the scope of this review, but a comprehensive review can be found here [64,65]. However, we will focus on CMV’s immunomodulatory proteins which potentially aid in viral dissemination. In order to infect innate immune cells, these cells must be attracted to the infection site. CMVs accomplish this through chemokine homologues. Chemokines are small activating and attracting proteins that generate a chemical gradient necessary for cellular chemotaxis [66,67]. It is hypothesized that CMVs recruit innate immune cells to the infection site in order to infect them and use them to egress from the primary infection site.

HCMV encodes two known CXC chemokine homologues and a potential CC chemokine homologue. *UL146* and *UL147* encode the CXC homologues, vCXCL-1 and vCXCL-2, respectively. While there is no functional data for vCXCL-2, vCXCL-1 has been extensively studied [68,69,70,71,72,73]. vCXCL-1 is a functional homologue of human chemokines CXCL8, CXCL1, and CXCL2, depending on the source of the vCXCL-1 protein. It signals primarily through the CXCR2 chemokine receptor, but with those with higher affinity of CXCR2 can also bind via CXCR1 as well [68,70]. A few potential CXCR2^+^ target cells include PMNs, inflammatory monocytes, and ECs [67,74,75,76,77]. Recently, Yamin et al. reported that vCXCL-1 can elicit a response through the CX3CR1 chemokine receptor [70]. Natural killer (NK) cells, which are CXCR1/CX3CR1 positive, responded to vCXCL-1 using both receptors [70]. While there is limited data as to whether NK cells are capable of harboring, replicating, or transferring HCMV, CXCR2^+^ cells can function as dissemination vehicles.

### 3.1. PMNs

PMNs (i.e., neutrophils) are the highest expressing CXCR2^+^ cells within the body. PMNs can harbor and transfer infectious HCMV [62,63,78]. In fact, the highest viral titers in the blood have been found within the PMN fraction [59,79]. Although PMNs are capable of harboring and transferring infectious HCMV, it is a non-productive infection [50,80]. These observations have been supported in a variety of animal models [24,81,82].

PMNs are rarely infected directly with cell-free virus [83]. Therefore, it appears that PMN infection is completely dependent on cell-to-cell spread [45,50]. HCMV transfer to PMNs is regulated by the presence of *UL146* and *UL147*. This implies that *UL146* and *UL147* may have additional functions besides chemotaxis [47]. After viral exposure, PMNs have an increased life span and express a pro-survival secretome [83]. Interestingly, PMNs exposed to cell-free virus release pro-inflammatory factors that induce monocyte recruitment and drive monocyte differentiation [83]. This could allow the neutrophil to “amplify” the immune response, which would bring in additional targets for infection and dissemination.

Two hypotheses could explain the role of PMNs in the course of primary CMV dissemination. One, PMNs directly disseminate HCMV or two, HCMV uses them indirectly. In the second scenario the neutrophil is recruited to the primary infection site where it encounters cell-free CMV. This interaction induces the PMN to secrete molecules that recruit other innate immune cells to the primary infection site [83]. These other immune cells are potentially better targets and will then be the primary CMV dissemination vehicle. Another role that PMNs might play during HCMV’s lifecycle is aiding dissemination after reactivation from latency. As reviewed in Reference [21], bone marrow is a major reservoir for latent HCMV [84]. Upon CXCR2 stimulation, neutrophils egress from the bone marrow [85]. Because vCXCL-1 has high affinity for CXCR2 [68,70], upon reactivation HCMV could use vCXCL-1 to stimulate neutrophils to leave the bone marrow and subsequently systemically disseminate HCMV. In this scenario, vCXCL-1’s main role is dissemination following reactivation from latency.

### 3.2. Monocytes and Macrophages

The monocyte is another myeloid-derived innate immune cell implicated in HCMV dissemination. Historically monocytes have been considered the major cell type for HCMV dissemination [2,3,86]. Monocytes are short-lived blood phagocytes that are precursors for inflammatory macrophages, inflammatory monocytes, and dendritic cells [87]. Like neutrophils, monocytes do not support productive HCMV replication [61,79,88,89]. However, productive monocyte infections have been reported once they have differentiated [90,91]. When naïve monocytes are exposed to either infectious HCMV or UV-inactivated virus they spontaneously undergo monocyte to macrophage differentiation [86,92]. These macrophages are capable of productive viral replication [93,94], so it seems logical that HCMV has evolved a mechanism for inducing monocyte to macrophage differentiation. Even though monocyte infection is not productive, they are capable of transferring infectious HCMV to uninfected cells in vitro [46]. The mechanism of this transfer is currently unknown. Infected monocytes also have reduced migration capacity as well as an impeded capacity to recruit other immune effectors, which would allow additional contact time to spread the virus as well as dampening immune activation [95].

**Figure 2:**
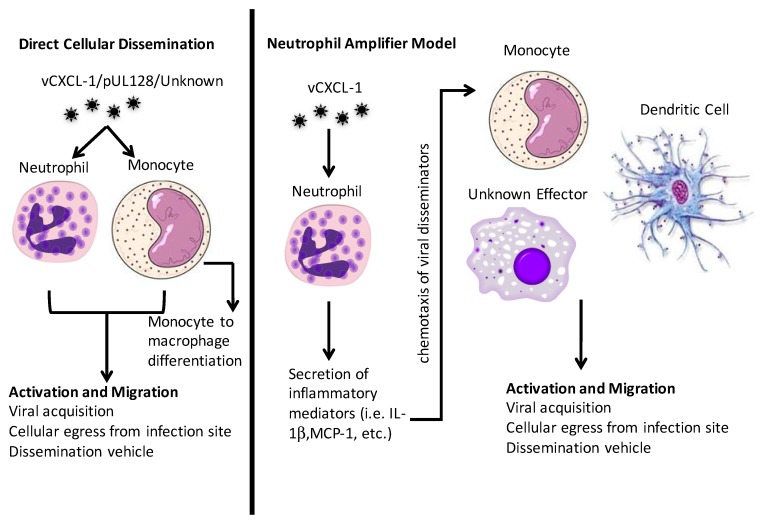
Graphical representation of direct cellular dissemination and the neutrophil amplifier model.

As with the HCMV vCXCL-1 for neutrophil recruitment, HCMV has evolved ways to attract monocytes to the infection site. HCMV encodes a CC chemokine homologue, pUL128. It is part of the pentameric entry complex, but purified pUL128 induces monocyte migration [96]. How this migration occurs and which chemokine receptor(s) is involved is unknown [97]. Because patrolling monocytes are CX3CR1 positive [87], it is possible that vCXCL-1 could elicit a monocyte response through this receptor. In addition, there are CXCR2^+^ monocytes [76,77,98] that could be responsive to vCXCL-1 as well. Monocytes are activated by and migrate towards CXCL8 (IL-8), to which vCXCL-1 is a functional homologue [77]. Therefore, HCMV could potentially elicit a monocytic response via a variety of different mechanisms and use these monocytes to aid in viral dissemination. Figure 2 summarizes the mechanisms that neutrophils and monocytes could play in viral dissemination.

## 4. Dissemination in Animal Models

Because the majority of HCMV infections are asymptomatic, studying primary dissemination in humans has been limited almost exclusively to in vitro and ex vivo analysis. A major characteristic of β-herpesviruses is their species specificity, meaning that HCMV is unable to productively replicate within another species [99,100,101]. Without the ability to use HCMV directly in animal models, animal CMVs have been used to study the mechanism of dissemination in vivo with these results extrapolated to HCMV characteristics and mechanisms. We will focus on small animal models of CMV infection because the majority of dissemination research has been carried out in them.

### 4.1. The Mouse Model

Predominantly MCMV has been used to draw conclusions about CMV dissemination. The mouse model is appealing for studying dissemination because MCMV has similar infection and pathogenesis to HCMV [102], MCMV contains homologues or orthologues, or both, of many HCMV genes, the mouse has a well-characterized immune system, and there are numerous reagents available including transgenic and knockout mice [103].

Like HCMV dissemination, innate immune cells mediate MCMV dissemination. MCMV encodes a potential functional homologue of *UL128* called *m131*. Both of these genes encode proteins that function as part of an entry complex and contain a CC chemokine motif [104]. The *m131* transcript spliced with *m129* form part of the entry complex referred to as MCK2. Like pUL128 [96], MCK2 has been shown to attract monocytes [105,106], pointing to the monocyte as a conserved dissemination vehicle across species. It is hypothesized that MCK2 functions through the chemokine receptor CX3CR1 as infection of mice lacking CX3CR1 on monocytes, dendritic cells, and NK cells [107] had greatly reduced viral dissemination to the salivary gland while primary dissemination was not impacted. It should be noted that when wild type mice were infected with mutant MCMV lacking MCK2, CX3CR1^+^ inflammatory monocytes were recruited to the infection site. However, the recruitment of CX3CR1^+^ patrolling monocytes was impeded. This indicates that the recruitment of all CX3CR1 innate immune cells is not solely reliant on MCK2. Therefore, it is likely that the initial systemic viral dissemination can progress without MCK2, but distal dissemination to the salivary gland is dependent on the recruitment of specific CX3CR1 positive immune cells [28]. This data further contradicts Fenner’s hypothesis. The MCMV secondary dissemination and primary dissemination are two independent events with potentially different cellular mediators as opposed to the sequential events which Fenner proposed.

Farrell et al. showed that dendritic cells (DCs) are responsible for salivary gland dissemination [27]. Both humans and mouse monocytes are capable of differentiating into DCs [108]. Therefore, it is possible that CX3CR1 monocytes are recruited to the infection site, become infected, and differentiate into DCs [109]. Although this differentiation of monocyte → DC could be the key, there could also be a different DC population that re-enters the circulation and is responsible for salivary gland dissemination [27]. Subsequent salivary gland infection and dissemination is also dependent on the MCMV M33 chemokine receptor homologue [27,110]. Likewise, HCMV has evolved a number of cytokine and chemokine receptor homologues, however their impact on dissemination has yet to be determined. A full review of these molecules and their functions or potential functions can be found in Reference [97].

While MCMV encodes a CC chemokine homologue it does not have a CXC chemokine homologue. This has limited the research on the role of neutrophils during MCMV infection. Recombinant MCMV’s overexpressing vCXCL-1 did not alter primary dissemination kinetics, however these viruses were impeded in their ability to efficiently infect the salivary gland [111]. These data point to a role of vCXCL-1 in dissemination, but overexpression of the chemokine induces an abnormal inflammatory environment, which halts normal salivary gland dissemination. Additionally, when neutrophils were depleted there was no significant impact on either primary or secondary dissemination implying that this effect is not neutrophil mediated [111]. Without a recombinant virus expressing vCXCL-1 under native conditions, it is difficult to discern the impact that neutrophils and the viral chemokine play in dissemination in this MCMV recombinant artificially expressing vCXCL-1, but point to differences between primary and secondary dissemination mechanisms.

The route of inoculation and immune control is often overlooked when studying dissemination. Mice are usually infected intraperitoneally, intravenously, or subcutaneously with MCMV [3]. However, infection via different inoculation routes yields different disease outcomes. For example, subcutaneous infection of CX3CR1-deficient mice limited salivary gland dissemination. When these same mice were infected intraperitoneally, the virus reached normal salivary gland titers [28]. When infecting mice intranasally (i.e., a more natural route), there was the expected cell-associated viremia but the virus did not infect the abdominal visceral organs (i.e., spleen, liver) [32]. This is unlike other infection routes where these organs are infected to relative high titers [24,28]. Therefore, in order to appropriately study dissemination in an animal model the route of inoculation should be taken into consideration.

In the mouse model, many of the immune cells that impact viral dissemination also function as viral controllers. This makes studying the cell types responsible for dissemination more difficult, even with all the tools the mouse model has to offer. In order to study the impact of specific innate immune cells on dissemination, immune cell populations can be depleted. The goal is to deplete the cells and measure an expected reduction in viral dissemination. Depletion experiments evaluating the monocytes’, macrophages’, and NK cells’ impact on dissemination resulted in exacerbated dissemination and increased viral burden within the organs [112,113,114]. This evidence highlights the importance of understanding that immune cells contribute multiple functions during the course of CMV infection and that they are not just vehicles for dissemination.

### 4.2. Other Small Animal Models

Dissemination in other small animal models has not been studied as in depth as MCMV. However, both rat CMV (RCMV) and guinea pig CMV (GPCMV) support observations from MCMV or ex vivo HCMV infections. Rat infection with RCMV showed that dissemination is reliant on infected PMNs and monocytic cells in the blood [81]. As with MCMV, RCMV’s *r129* and *r131* which also encode CC chemokines. R129 protein induces macrophage, PBMC, and lymphocyte (more specifically naïve CD4^+^ T cell) migration and activation and increases transplant vascular sclerosis [115]. r131 increases the number of macrophages at the infection site and is needed to efficiently disseminate to the salivary gland [116]. The RCMV chemokine receptor, R33, functions similar to its counterpart M33 in MCMV. R33 plays an important role in inducing inflammatory responses that contribute to viral dissemination and accelerates transplant rejection [117,118].

The GPCMV gene, *gp1*, encodes a CC chemokine as well. This chemokine, GPCMV-MIP, [119] is similar to human MIP-1 (macrophage inflammatory protein-1) and binds human CCR1 [120]. Knocking out *gp1* in the GPCMV genome resulted in decreased inflammation and reduced hearing loss in pups compared with those infected with WT virus [121,122]. When mice were infected with a *gp1* knockout virus, it served as an attenuated vaccine, which inhibited transplacental transmission of GPCMV [123]. These results highlight the importance of viral chemokines in CMV spread and viral pathogenesis as well as its potential use in generating an attenuated vaccine.

GPCMV has also furthered our understanding of the pentameric entry complex. Recombinant GPCMV lacking GP129-GP133—the HCMV pentameric complex homologue—had impaired cellular entry and failed to spread in vivo [124]. GPCMV infection of guinea pigs is the best small animal model to study transplacental transmission of CMVs. MCMV and RCMV do not efficiently cross the placenta and infect offspring in utero, but GPCMV does. An extensive review of this model and its uses for vaccine and drug development can be found in References [125,126].

## 5. Conclusions

**Figure 3:**
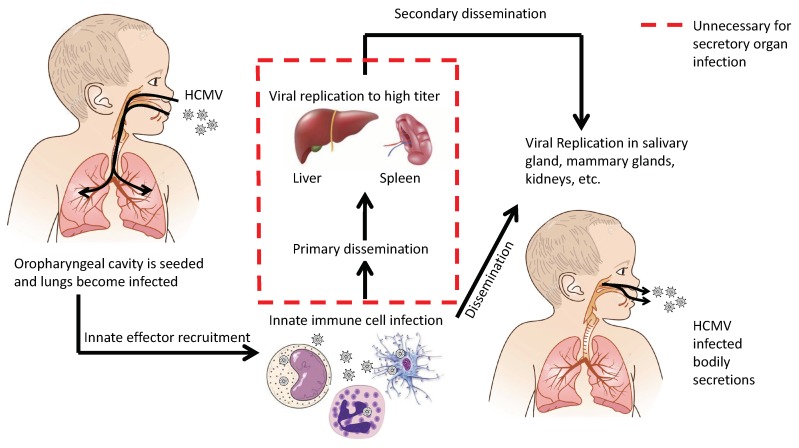
Alternative mechanism of HCMV dissemination generated from animal model extrapolation.

Dissemination of cytomegaloviruses has two goals: (1) achieve systemic dissemination in order to be transmitted to a new host and (2) establish latency within the current host. In either case, there are multiple mechanisms to achieve these goals. There are key events and cell types that impact dissemination. First, infection occurs in the oropharyngeal cavity, resulting in upper respiratory tract infection. These cells then release infectious viral progeny, viral chemotactic factors, and induce the innate responses. This environment will induce the invasion of innate immune cells into the tissue. These immune cells become infected and traffic the virus throughout the body. Figure 3 illustrates an alternative mechanism of systemic dissemination and modifies Fenner’s original sequential dissemination (i.e., infection site → draining lymph nodes → blood filtering organs) [22,23]. We propose an alternative mechanism for CMV dissemination (Figure 3) where virally infected cells can directly seed secondary organs that lead to secretion in bodily fluids. As we have seen time and time again, CMV deviates from the norms of viral infections and, as always, has its own agenda.
